# Supplementary material for: High school science fair: Experiences of two groups of undergraduate bioscience students
Source: PLoS One. 2021 Jun 4;16(6):e0252627. doi: 10.1371/journal.pone.0252627 (PMC8177480; doi:10.1371/journal.pone.0252627)
Supplement: S1 Survey — (PDF) [file pone.0252627.s003.pdf]

# UTSW Students -- Science Fair Survey

We are interested in learning about student experience with high school science fair. Even if you haven't done science fair, we would like to know about your general impressions concerning what help it would be reasonable for students to receive when doing science fair projects and what obstacles students might face. This survey, which consists of up to about 20 questions, is anonymous and should only require about 10 minutes to complete. We only are interested in overall trends. You may leave blank any question you prefer not to answer. It is important that you give honest replies to those questions that you do answer since the results may be used to influence science fair practices in the future. Your participation in the survey is voluntary; however, the more students who participate in the survey, the more statistically persuasive will be any conclusions.

1. I am a UTSW
   - ◯ MD Student
   - ◯ MD-PhD Student
   - ◯ PhD Student

2. Gender?
   - ◯ Female
   - ◯ Male

3. When you were in high school, did you carry out a science fair project?
   - ◯ Yes
   - ◯ No
   - ◯ I am unfamiliar with science fair

4. Was carrying out a science fair project possible at your high school?
   - ◯ Yes
   - ◯ No

5. What was the reason that you did not carry out a science fair project?
Check all that apply.
   - ☐ 1. Not interested
   - ☐ 2. Not enough time
   - ☐ 3. Too expensive
   - ☐ 4. Not enough help available
   - ☐ 5. Did not have a good idea for a project
   - ☐ 6. Did not want to compete
   - ☐ 7. Other

Specify: ______________________________

4. If you carried out science fair in high school, then what was the highest grade in which you did so?
   - ◯ 9th
   - ◯ 10th
   - ◯ 11th
   - ◯ 12th

4a. Did you carry out science fair more than once?
   - ◯ Yes
   - ◯ No

If you carried out science fair more than once, then on subsequent questions use your most recent experience to answer.

5. Was your science fair project Team or Individual?
   - ◯ Team
   - ◯ Individual

6. Was the science fair project required by your school?
   - ◯ Yes
   - ◯ No
   - ◯ No, but I did a science fair project to satisfy a school project requirement.

7. Do you think science fair projects should be optional or required? (This need not be for competition.)
   - ◯ Optional
   - ◯ Required

8. Reason why?

______________________________

9. Do you think science fair projects for competition should be optional or required?
   - ◯ Optional
   - ◯ Required

10. Reason why? __________________________________

11. From whom do you think it would be reasonable to receive help on a science fair project?
Check all that apply

- ☐ 1. Parents
- ☐ 2. Siblings
- ☐ 3. Other family members (uncles, cousins, etc.)
- ☐ 4. Teachers
- ☐ 5. Other students
- ☐ 6. Scientists
- ☐ 7. A paid mentor
- ☐ 8. Articles on the Internet
- ☐ 9. Articles in books or magazines
- ☐ Other

Specify: ____________________________

12. Who actually helped you?
Check all that apply

- ☐ 1. Parents
- ☐ 2. Siblings
- ☐ 3. Other family members (uncles, cousins, etc.)
- ☐ 4. Teachers
- ☐ 5. Other students
- ☐ 6. Scientists
- ☐ 7. A paid mentor
- ☐ 8. Articles on the Internet
- ☐ 9. Articles in books or magazines
- ☐ Other

Specify: ____________________________

13. What kind of help on a science fair project do you think would be reasonable to expect from others?
Check all that apply

- ☐ 1. Being given the main idea
- ☐ 2. Development of the idea
- ☐ 3. Gathering background research information, or finding a research site or participants
- ☐ 4. Performing the experiments
- ☐ 5. Writing the report
- ☐ 6. Fine tuning the report after it is written
- ☐ 7. Designing the poster board and presentation
- ☐ 8. Producing charts or graphs
- ☐ 9. Coaching for the interview with judges
- ☐ 10. Copying the project from someone else
- ☐ Other

Specify? ____________________________

14. What kind of help did you actually receive?
Check all that apply

- ☐ 1. Being given the main idea
- ☐ 2. Development of the idea
- ☐ 3. Gathering background research information, or finding a research site or participants
- ☐ 4. Performing the experiments
- ☐ 5. Writing the report
- ☐ 6. Fine tuning the report after it is written
- ☐ 7. Designing the poster board and presentation
- ☐ 8. Producing charts or graphs
- ☐ 9. Coaching for the interview with judges
- ☐ 10. Copying the project from someone else
- ☐ Other

Specify: ____________________________

15. Did you get the kind of help you wanted from teachers?

- ◯ Yes
- ◯ No

16. Was there some kind of help that you would have liked but did not receive?
Specify: ________________________________

17. Did you get the amount of help you wanted from teachers?

- ◯ Yes
- ◯ No

18. Were the results of your project as expected?

- ◯ Yes
- ◯ No

19. What obstacles did you face?
Check all that apply

- ☐ 1. Coming up with the main idea
- ☐ 2. Getting motivated to do the project
- ☐ 3. Becoming disappointed  with the project
- ☐ 4. Limited resources
- ☐ 5. Limited knowledge
- ☐ 6. Limited skills
- ☐ 7. Limited cooperation
- ☐ 8. Getting organized
- ☐ 9. Time pressure
- ☐ 10. Not enough money
- ☐ 11. Results not as expected
- ☐ Other

Specify? ________________________________

19. What obstacles do you think students who do science fair usually face?
Check all that apply

- ☐ 1. Coming up with the main idea
- ☐ 2. Getting motivated to do the project
- ☐ 3. Becoming disappointed  with the project
- ☐ 4. Limited resources
- ☐ 5. Limited knowledge
- ☐ 6. Limited skills
- ☐ 7. Limited cooperation
- ☐ 8. Getting organized
- ☐ 9. Time pressure
- ☐ 10. Not enough money
- ☐ 11. Results not as expected
- ☐ Other

Specify? ________________________________

20. How did you overcome the obstacles you encountered?
Check all that apply

- ☐ 1. Used someone else's main idea
- ☐ 2. Picked a familiar/interesting topic
- ☐ 3. Did more background research
- ☐ 4. Stopped working on the project for a while
- ☐ 5. Made a timeline to follow
- ☐ 6. Perseverance and self-discipline
- ☐ 7. Had someone else to keep me on track
- ☐ 8. Had someone else do the math
- ☐ 9. Changed the research plan
- ☐ 10. Collected more data
- ☐ 11. Had someone else collect the data
- ☐ 12. Used someone else's data
- ☐ 13. Made up the data
- ☐ 14. Changed the hypothesis to fit the data
- ☐ 15. Changed the data to fit the hypothesis
- ☐ Other

Specify? ________________________________

20. How do you think students who do science fair usually overcome obstacles?
Check all that apply

- ☐ 1. Use someone else's main idea
- ☐ 2. Pick a familiar/interesting topic
- ☐ 3. Do more background research
- ☐ 4. Stop working on the project for a while
- ☐ 5. Make a timeline to follow
- ☐ 6. Perseverance and self-discipline
- ☐ 7. Have someone else to keep them on track
- ☐ 8. Have someone else do the math
- ☐ 9. Change the research plan
- ☐ 10. Collect more data
- ☐ 11. Have someone else collect the data
- ☐ 12. Use someone else's data
- ☐ 13. Make up the data
- ☐ 14. Change the hypothesis to fit the data
- ☐ 15. Change the data to fit the hypothesis
- ☐ Other

Specify? ______________________________
