# Supplementary figures and images for: High school science fair: Experiences of two groups of undergraduate bioscience students
Source: PLoS One. 2021 Jun 4;16(6):e0252627. doi: 10.1371/journal.pone.0252627 (PMC8177480; doi:10.1371/journal.pone.0252627)

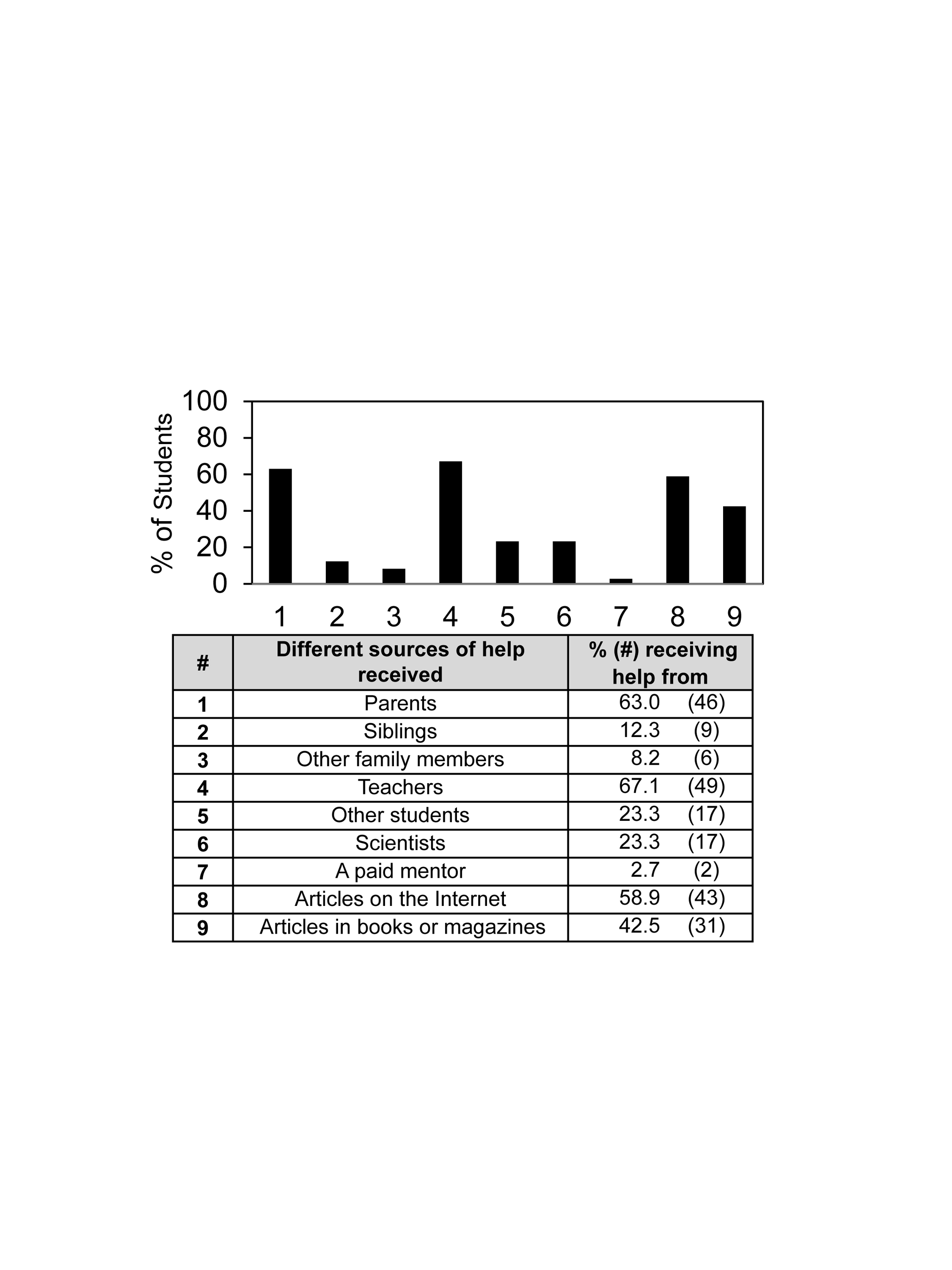

Supplement: S1 Fig — (TIF) [file pone.0252627.s004.tif]

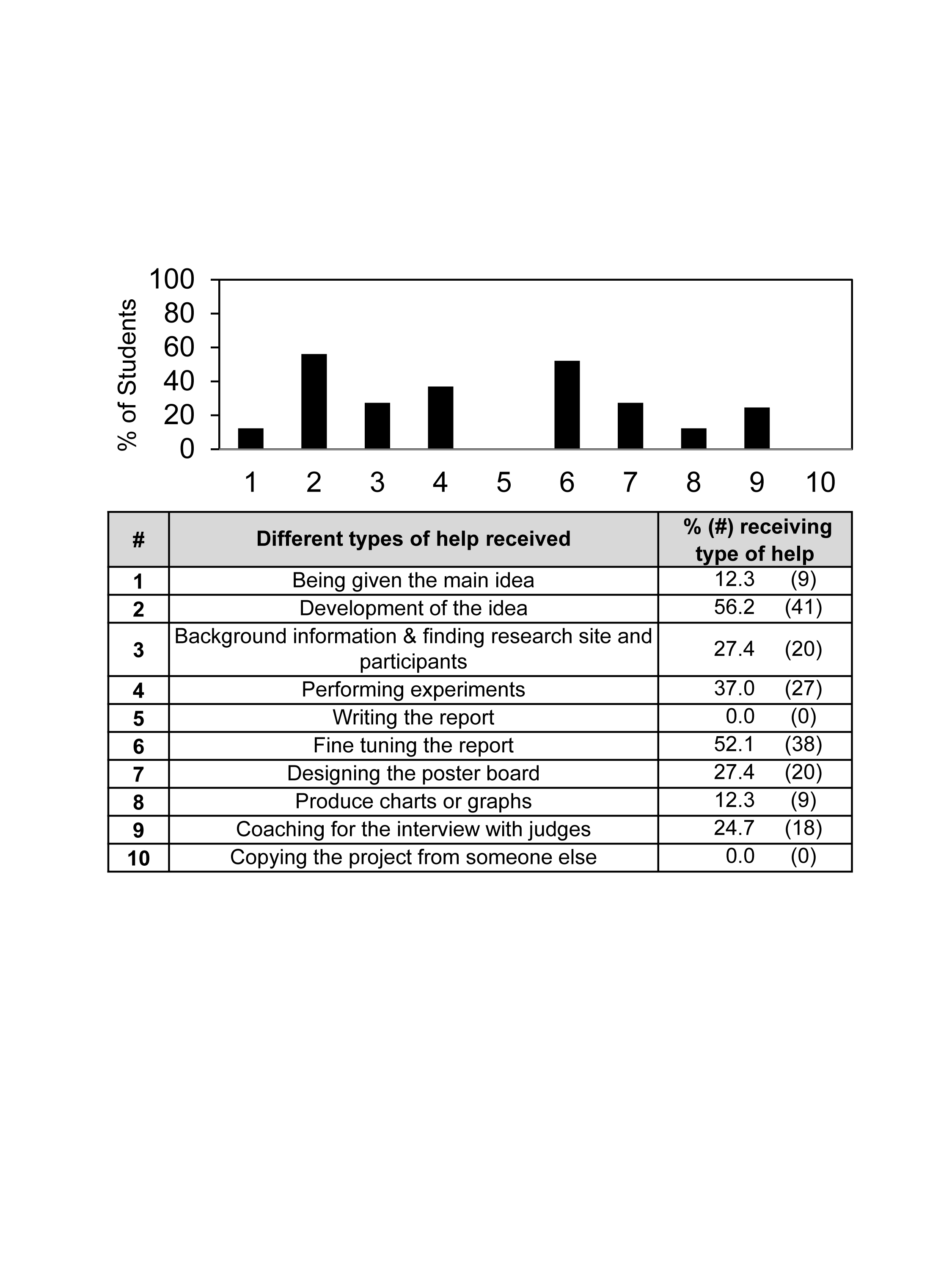

Supplement: S2 Fig — (TIF) [file pone.0252627.s005.tif]

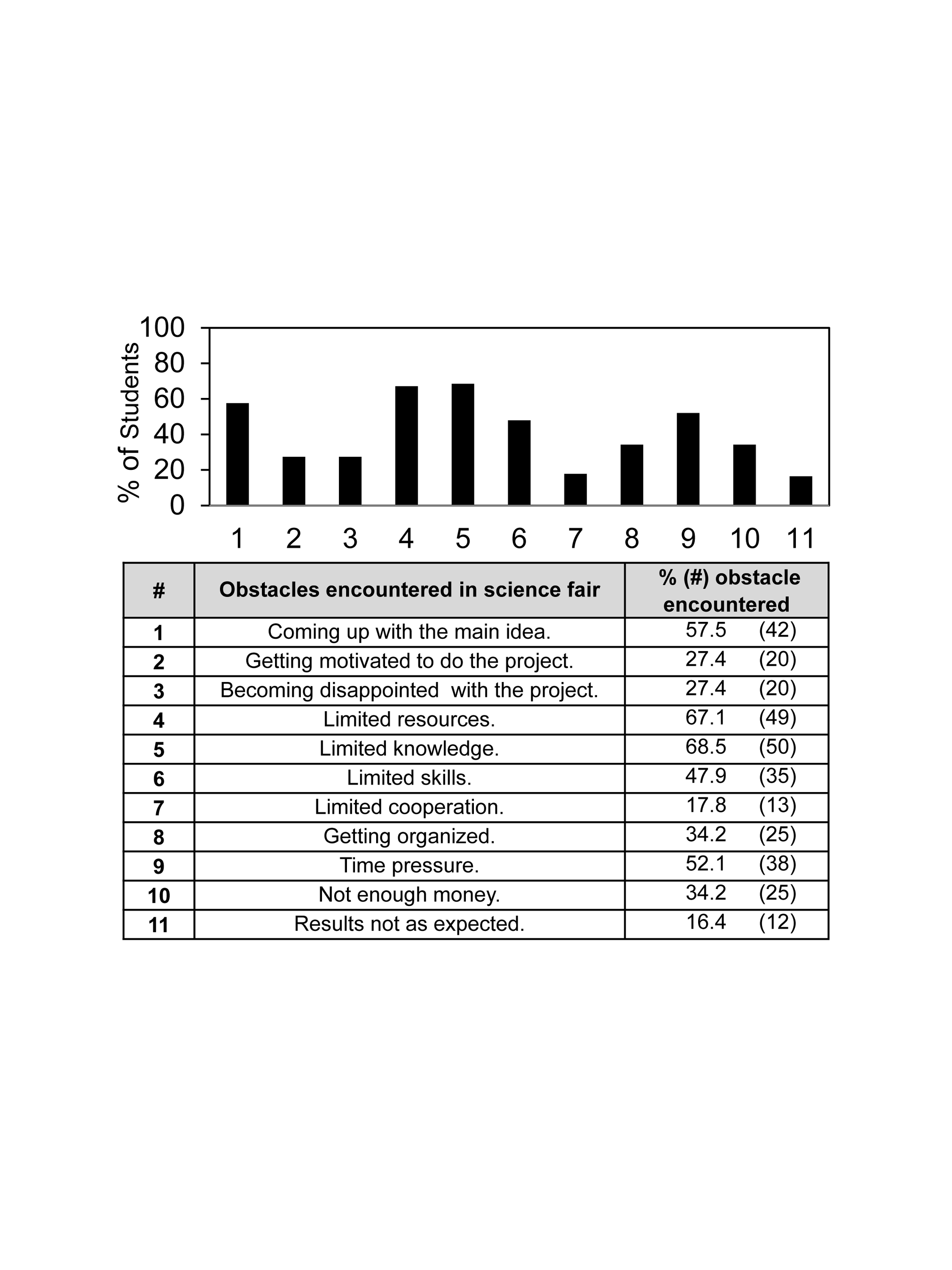

Supplement: S3 Fig — (TIF) [file pone.0252627.s006.tif]

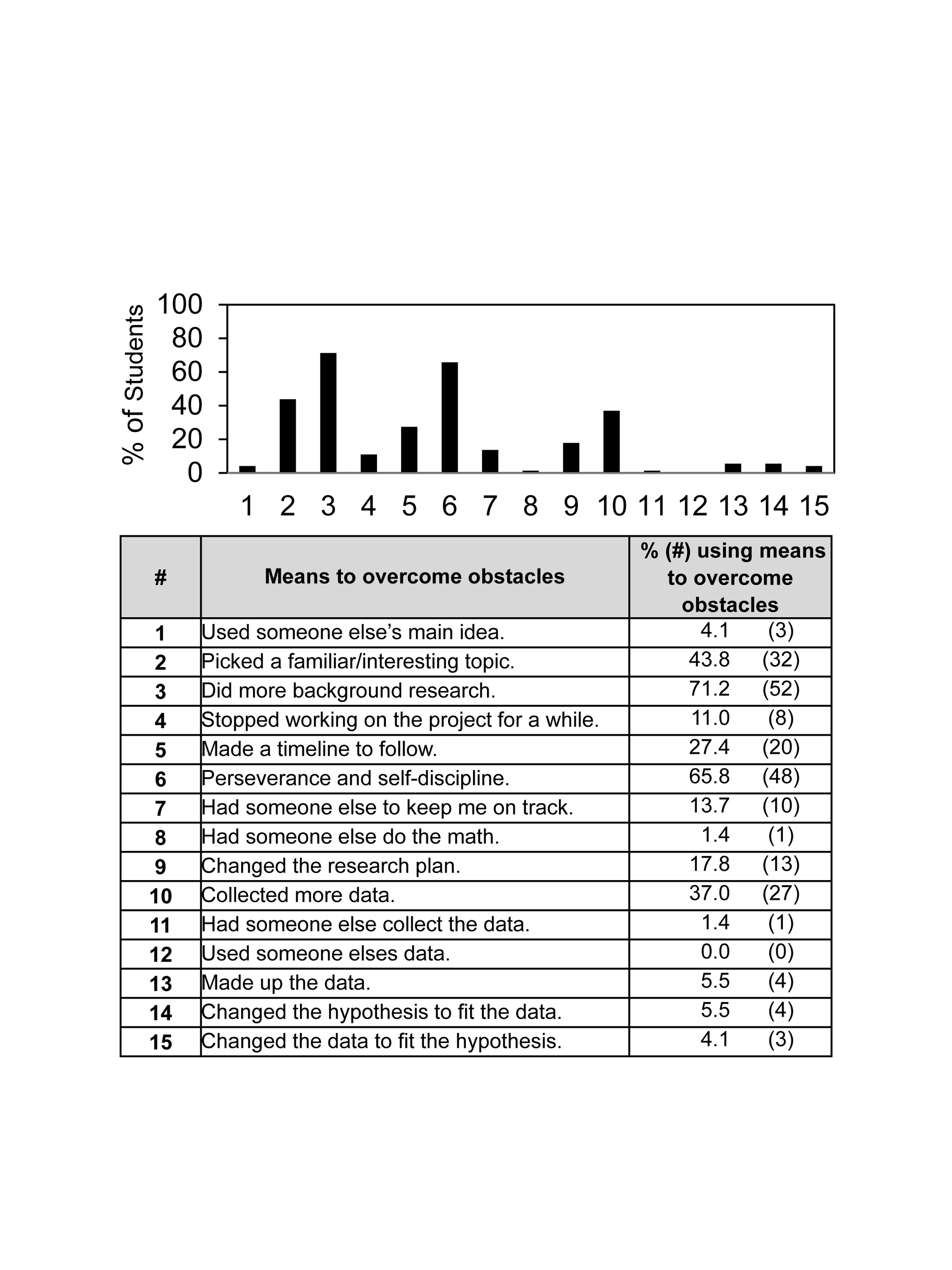

Supplement: S4 Fig — (TIF) [file pone.0252627.s007.tif]
